# Supplementary material for: Temporal assortment of cooperators in the spatial prisoner’s dilemma
Source: Commun Biol. 2021 Nov 12;4:1283. doi: 10.1038/s42003-021-02804-9 (PMC8589994; doi:10.1038/s42003-021-02804-9)
Supplement: Supplementary file 2 — Reporting Summary [file 42003_2021_2804_MOESM2_ESM.pdf]

nature research

Corresponding author(s): Tim Johnson

Last updated by author(s): Sep 19, 2021

Reporting Summary

Nature Research wishes to improve the reproducibility of the work that we publish. This form provides structure for consistency and transparency in reporting. For further information on Nature Research policies, see our Editorial Policies and the Editorial Policy Checklist.

Statistics

For all statistical analyses, confirm that the following items are present in the figure legend, table legend, main text, or Methods section.

n/a	Confirmed	
☒	☐	The exact sample size (*n*) for each experimental group/condition, given as a discrete number and unit of measurement
☒	☐	A statement on whether measurements were taken from distinct samples or whether the same sample was measured repeatedly
☒	☐	The statistical test(s) used AND whether they are one- or two-sided<br>*Only common tests should be described solely by name; describe more complex techniques in the Methods section.*
☒	☐	A description of all covariates tested
☒	☐	A description of any assumptions or corrections, such as tests of normality and adjustment for multiple comparisons
☒	☐	A full description of the statistical parameters including central tendency (e.g. means) or other basic estimates (e.g. regression coefficient) AND variation (e.g. standard deviation) or associated estimates of uncertainty (e.g. confidence intervals)
☒	☐	For null hypothesis testing, the test statistic (e.g. F, t, r) with confidence intervals, effect sizes, degrees of freedom and P value noted<br>*Give P values as exact values whenever suitable.*
☒	☐	For Bayesian analysis, information on the choice of priors and Markov chain Monte Carlo settings
☒	☐	For hierarchical and complex designs, identification of the appropriate level for tests and full reporting of outcomes
☒	☐	Estimates of effect sizes (e.g. Cohen's d, Pearson's r), indicating how they were calculated

Our web collection on statistics for biologists contains articles on many of the points above.

Software and code

Policy information about availability of computer code

Data collection	Data studied in the paper were generated in simulations written in Python 3.7.6. All computer code is available online via a project page hosted by the Open Science Framework (DOI: 10.17605/OSF.IO/3JSXV).
Data analysis	Data generated in the simulations were analyzed in R 3.5.3. All computer code is available online via a project page hosted by the Open Science Framework (DOI: 10.17605/OSF.IO/3JSXV).

For manuscripts utilizing custom algorithms or software that are central to the research but not yet described in published literature, software must be made available to editors and reviewers. We strongly encourage code deposition in a community repository (e.g. GitHub). See the Nature Research guidelines for submitting code & software for further information.

Data

Policy information about availability of data

All manuscripts must include a data availability statement. This statement should provide the following information, where applicable:

- Accession codes, unique identifiers, or web links for publicly available datasets
- A list of figures that have associated raw data
- A description of any restrictions on data availability

Data sets used in the study are publicly available online via a project page hosted by the Open Science Framework (DOI: 10.17605/OSF.IO/3JSXV). Computer code provided at that same project page can be used to reproduce all figures in this paper using the data supplied on the project page.

Field-specific reporting

Please select the one below that is the best fit for your research. If you are not sure, read the appropriate sections before making your selection.

☐ Life sciences ☐ Behavioural & social sciences ☒ Ecological, evolutionary & environmental sciences

For a reference copy of the document with all sections, see nature.com/documents/nr-reporting-summary-flat.pdf

Ecological, evolutionary & environmental sciences study design

All studies must disclose on these points even when the disclosure is negative.

Study description	The study involved a computer simulation of an artificial population, with the complete computer code provided via a project page hosted by the Open Science Framework (DOI: 10.17605/OSF.IO/3JSXV).
Research sample	Computer-generated population of simulated agents.
Sampling strategy	N/A
Data collection	Data generated via simulation.
Timing and spatial scale	N/A
Data exclusions	N/A
Reproducibility	Computer code is available for inspection and can be accessed via a project page hosted by the Open Science Framework (DOI: 10.17605/OSF.IO/3JSXV).
Randomization	Computer-generated numerical randomization in the simulation.
Blinding	Given that the authors of the simulation also performed the data analysis and given that data output could not be blinded in the simulation without risk of mis-identifying simulation output (i.e. variable names needed to be known to conduct analyses), blinding was not possible.

Did the study involve field work? ☐ Yes ☒ No

Reporting for specific materials, systems and methods

We require information from authors about some types of materials, experimental systems and methods used in many studies. Here, indicate whether each material, system or method listed is relevant to your study. If you are not sure if a list item applies to your research, read the appropriate section before selecting a response.

Materials & experimental systems

n/a	Involved in the study	
☒	☐	Antibodies
☒	☐	Eukaryotic cell lines
☒	☐	Palaeontology and archaeology
☒	☐	Animals and other organisms
☒	☐	Human research participants
☒	☐	Clinical data
☒	☐	Dual use research of concern

Methods

n/a	Involved in the study	
☒	☐	ChIP-seq
☒	☐	Flow cytometry
☒	☐	MRI-based neuroimaging
